# Medical Record Abstraction for Quality Improvement in Sepsis Care Using Artificial Intelligence: A Cluster Randomized Trial

**DOI:** 10.1001/jamanetworkopen.2026.11885

**Published:** 2026-06-25

**Authors:** Aaron Boussina, Claire Allison, Kimberly Quintero, Sonia Jain, Chad VanDenBerg, Michael Hogarth, Amy M. Sitapati, Karandeep Singh, Atul Malhotra, Michael T. McCurdy, Christopher A. Longhurst, James S. Ford, Theodore Chan, Paul Ishimine, Richard Childers, Shamim Nemati, Gabriel Wardi

**Affiliations:** 1 Division of Biomedical Informatics, University of California, San Diego, San Diego, California; 2 Joan & Irwin Jacobs Center for Health Innovation, University of California, San Diego, San Diego; 3 School of Medicine, University of California, San Diego, San Diego; 4 Department of Quality, University of California, San Diego, San Diego; 5 Department of Family Medicine and Public Health, University of California, San Diego, San Diego; 6 Division of Pulmonary, Critical Care and Sleep Medicine, University of California, San Diego, San Diego; 7 Division of Pulmonary and Critical Care Medicine, University of Maryland School of Medicine, Baltimore; 8 Department of Emergency Medicine, University of California, San Diego, San Diego

## Abstract

**Importance:**

Hospital quality reporting remains a manual, costly process with critical limitations as a mechanism to improve care outcomes.

**Objective:**

To assess whether near-real-time quality measurement, enabled by large language models (LLMs), can improve quality performance as measured by the Centers for Medicare & Medicaid Services (CMS) Severe Sepsis and Septic Shock Management Bundle (SEP-1) quality metric.

**Design, Setting, and Participants:**

This single-blind, unstratified, cluster randomized trial was conducted between December 13, 2024, and July 8, 2025, at 2 academic emergency departments (EDs) within the University of California, San Diego (UCSD) health system. Participants included all 66 attending physicians who practiced in the UCSD EDs and worked more than 3 shifts per month prior to study initiation.

**Intervention:**

Participants were randomized to receive targeted feedback from LLM-determined compliance with SEP-1 at the time of patient discharge or standard process.

**Main Outcomes and Measures:**

The primary outcome was overall compliance with SEP-1. Secondary outcomes included expert agreement with the LLM SEP-1 determination, 30-day mortality, and intensive care unit admissions of patients with severe sepsis and/or septic shock in the ED. Effect sizes were estimated from a mixed-effects logistic regression model with the intervention group as a fixed effect and a random intercept for physician.

**Results:**

The study population included 66 physicians who treated 301 patients (121 in the control group and 180 in the intervention group; median age, 64.3 [IQR, 51.1-75.7] years; 171 [56.8%] male; 52 [17.3%] with chronic kidney disease; 52 [17.3%] with chronic heart failure) who met CMS inclusion criteria for SEP-1. Physicians in the control group had a SEP-1 compliance rate of 70.1%, while those in the intervention group had a rate of 82.9%. Assignment to the intervention group resulted in a 13.0% absolute improvement in SEP-1 compliance (95% CI, 2.5%-23.4%; odds ratio, 2.10 [95% CI, 1.15-3.81]; *P* = .02) in the mixed-effects model. The largest difference between the intervention group and control group was in noncompletion of the 30-mL/kg fluid bolus component (3 of 180 [1.7%] vs 16 of 121 [13.2%]), a documentation-sensitive component of the quality measure. Agreement between LLM determination and expert review was 92%. No significant differences existed in intensive care unit admissions or 30-day mortality.

**Conclusions and Relevance:**

In this cluster randomized trial of artificial intelligence (AI)-enabled medical record abstraction for sepsis care, rapid assessment of SEP-1 performance and targeted feedback improved overall compliance with the measure. AI-driven quality clinical integration may address limitations in existing hospital quality reporting and better support a learning health system.

**Trial Registration:**

ClinicalTrials.gov Identifier: NCT07581340

## Key Points

**Question:**

Can automated assessment of a complex care quality measure with more timely feedback improve measure performance?

**Findings:**

In this cluster randomized trial of 66 physicians treating 301 patients, the use of a large language model to automatically assess a complex measure for severe sepsis and septic shock and deliver targeted feedback improved quality metric performance. No change in 30-day mortality was observed among patients with sepsis cared for by physicians in the control and intervention groups.

**Meaning:**

These findings suggest that artificial intelligence may enable quality clinical integration to address limitations in current quality measurement.

## Teaser

This cluster randomized trial assesses whether the receipt of targeted feedback based on artificial intelligence (AI)-enabled medical record abstraction can improve physician compliance with the Centers for Medicare & Medicaid Services Severe Sepsis and Septic Shock Management Bundle.

## Introduction

In a 2001 landmark report,^1^ the National Academy of Medicine highlighted the pressing need to simplify quality measurement, the evaluation of care, and improve its dissemination into clinical practice to support safe, effective, patient-centered, timely, efficient, and equitable health care. However, while progress has been made to decrease hospital-acquired infections, perioperative care, and ultimately patient-centered outcomes in the past quarter century, major gaps remain.^2,3,4,5,6,7^ For example, 1 study estimated that quality reporting for a single acute care hospital required more than 100 000 person-hours, costing over $5 million annually.^8^ Furthermore, quality measurement is often too untimely to be actionable and too underpowered to be statistically valid.^9,10^

These challenges are well reflected in the Severe Sepsis and Septic Shock Management Bundle (SEP-1) reported to the Centers for Medicare & Medicaid Services (CMS).^11^ This complex, 63-step measure has remained controversial in part due its manual and costly abstraction with poor interrater reliability.^12,13,14^ However, the measure remains an important component of sepsis care across the US, with direct financial ramifications due to its recent addition to the CMS Hospital Value-Based Purchasing Program.^15^ A previous report that included several of the present investigators^16^ found that large language models (LLMs) can accurately abstract complex quality measures such as SEP-1 from unstructured clinical documentation, which may address prior concerns. For example, at the University of California, San Diego (UCSD), an artificial intelligence (AI)−based human-in-the-loop workflow recently replaced the existing SEP-1 reporting process involving medical record review by an external contractor (Nicholas Hilbert; K.Q., C.V., et al; unpublished data, September 15, 2025). While this addresses challenges related to cost and manual variability, the CMS mandates reporting on only 20 cases a month, 4 months after discharge, across our health system. As sepsis remains the leading cause of mortality and morbidity worldwide, we posit that SEP-1 measurement at this scale is suboptimal for driving improvement in measure performance.^17^

In this study, we sought to test the hypothesis that AI-enabled scaling of SEP-1 measurement beyond standard sampling and near–real-time communication of care quality performance by individual physicians can improve measure performance. We selected SEP-1 due to its well-described complexity, its proportionally small sampling, its financial importance to health systems, and its significant global health burden.

## Methods

### Study Design and Setting

We conducted a prospective, cluster randomized trial between December 13, 2024, and July 8, 2025, in the emergency departments (EDs) of 2 academic medical centers within the UCSD health system (Hillcrest Medical Center, San Diego, and Jacobs Medical Center, La Jolla). The Aligning and Coordinating Quality Improvement, Research, and Evaluation Committee deemed the study exempt from ethics approval and the need for informed consent owing to its quality improvement study design.^18^ The study followed the Consolidated Standards of Reporting Trials Extension (CONSORT Extension) reporting guideline. The trial protocol and statistical analysis plan are provided in Supplement 1. The trial was not prospectively registered at ClinicalTrials.gov because it was developed as a quality improvement study; it was registered retrospectively at the request of the journal editors.

**Figure 1.**
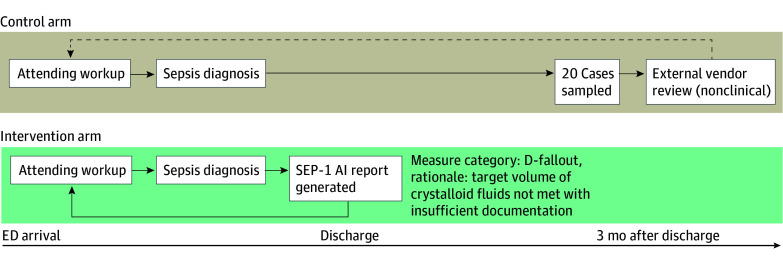
Flow Diagram of Artificial Intelligence (AI)–Driven Learning Health System Intervention A minority of patients with sepsis attended to by physicians in the control group were selected for external review 3 months after discharge as part of standard quality reporting. All patients with sepsis attended to by physicians in the intervention group were evaluated via generative AI with active feedback at the time of discharge. ED indicates emergency department; SEP-1, Centers for Medicare & Medicaid Services Severe Sepsis and Septic Shock Management Bundle. Fallout indicates a noncompliant case.

All 66 attending emergency medicine physicians working at least 3 ED shifts per month were randomized to either (1) receive timely feedback on the quality of sepsis care using the LLM (intervention group) or (2) follow the standard approach to feedback on sepsis quality (control group) (Figure 1). Most patient care (>95%) at the Hillcrest Medical Center ED is provided by residents under attending physician supervision, whereas approximately 50% of patient care at the Jacobs Medical Center ED is provided by either a resident or advanced practice clinician under attending physician supervision. The remainder of patients are seen primarily by attending physicians, without any involvement from residents or advanced practice clinicians. Randomization was single blind and unstratified. Patients with sepsis included for review were identified using the same criteria for severe sepsis and septic shock as set forth by CMS and adjudicated according to this metric as pass, fail, or out of measure. The clinical encounter diagnosis was used instead of the billing diagnosis because the latter is often unavailable at the time of discharge. Following study completion, we extracted the billing diagnoses from electronic health records (Epic Clarity; Epic Systems Corporation) to evaluate agreement between the clinical diagnosis and standard delayed eligibility criteria for SEP-1. All patients with a time zero (defined by CMS) of severe sepsis and/or septic shock under the care of an attending emergency physician were included in the study and were automatically evaluated for SEP-1 compliance using our LLM system at the time of discharge.

Patients self-reported their race as Asian, Black or African American, White, or multiracial or other (including American Indian or Alaska Native, Native Hawaiian or Other Pacific Islander, or known). Ethnicity was self-reported as Hispanic, Latina, Latino, or of Spanish origin; not Hispanic, Latina, Latino, or of Spanish origin; or unknown. These data were collected to better contextualize the patient mix.

### LLM Architecture

The architecture and performance of this system have been previously described with 90% agreement with expert human reviewers.^16^ In our study, we used Llama, version 3.1 8B instruct LLM (Meta Platforms Inc; July 23, 2024, release) because it is a small model fine-tuned to follow instructions, making it suitable for performing specific abstraction tasks provided in the prompt.^16,19^ The prompts used have been reported previously.^16,20^ We performed test-time scaling of 3 runs with majority voting to mitigate hallucinations.^21^

With a baseline 65% SEP-1 compliance, a sample size of 300 patients would be required to identify a 15% increase in compliance in the intervention group with a power of 0.8 and a significance level of .05. Three study team members with extensive experience in SEP-1 adjudication (C.A., K.Q., and G.W.) reviewed each in-measure case for agreement with the system, and a final decision on SEP-1 compliance was made. Iterative improvements to the system were made based on adjudication of cases (eTable 1 in Supplement 2). If the care of a physician in the intervention group met all elements of SEP-1, they received a standardized congratulatory email and a brief reminder of UCSD’s sepsis policy. Physicians whose patient care did not meet the metric were sent an email summary of the case and occasionally received a phone call from the medical director of our hospital’s sepsis program (G.W.) for targeted feedback in the email that included the reason for noncompliance and how to improve care or documentation. If multiple reasons for noncompliance were present, each of these was highlighted to the physician. No graphics were included in the feedback. Physicians randomized to the usual care control group received feedback via the same approach, but only if a case of sepsis was sent to the CMS for public reporting per standard random sampling of 20 cases a month, typically 3 to 4 months after discharge, across ED and inpatient settings.

### Statistical Analysis

Our primary outcome was SEP-1 compliance as defined by the CMS. Secondary outcomes included agreement between the system and human reviewers, 30-day mortality, and intensive care unit admissions of patients with severe sepsis and/or septic shock in the ED during the quality improvement period. Because randomization occurred at the physician level and outcomes were measured at the patient encounter level, analyses accounted for clustering of encounters within physicians. The primary analysis used a mixed-effects logistic regression model with SEP-1 compliance as the dependent variable, intervention group as a fixed effect, and a random intercept for physician. We reported the corresponding odds ratio from the model and estimated the effect of the intervention on compliance using the model-based mean marginal effect. We repeated this with all secondary outcomes as dependent variables. We further conducted a secondary analysis of physician-level comparisons of compliance rates using a *t* test. Analyses were conducted using R, version 4.5.2 (lme4 version 1.1.38; performance version 0.16.0 [R Project for Statistical Computing]). Two-sided α < .05 was considered statistically significant in all analyses.

## Results

**Figure 2.**
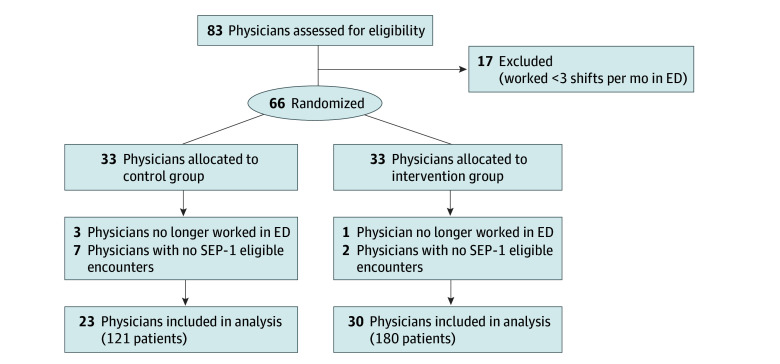
CONSORT Flow Diagram ED indicates emergency department; SEP-1, Centers for Medicare & Medicaid Services Severe Sepsis and Septic Shock Management Bundle.

Our study population included 301 patients (121 in the control group and 180 in the intervention group; median age, 64.3 [IQR, 51.1-75.7] years; 130 [43.2%] female and 171 [56.8%] male; 52 [17.3%] with chronic kidney disease; 52 [17.3%] with chronic heart failure) who met CMS inclusion criteria for SEP-1 (Table 1). In terms of race, 33 patients (11.0%) were Asian, 26 (8.6%) were Black or African American, 141 (46.8%) were White, and 101 (33.6%) were multiracial or of other race. In terms of ethnicity, 106 patients (35.2%) were Hispanic, Latina, Latino, or of Spanish origin; 192 patients (63.8%) were not Hispanic, Latina, Latino, or of Spanish origin; and 3 patients (1.0%) did not report their ethnicity. Of 66 eligible attending physicians, 33 were randomized to the intervention group and 33 were randomized to the control group (Figure 2). After randomization occurred, 1 physician in the intervention group and 3 in the control group had a change in clinical duties and no longer worked in the ED. Additionally, 2 physicians in the intervention group and 7 in the control group had no SEP-1 eligible encounters and were therefore excluded from the final analysis.

**Table 1.** Patient Characteristics

Characteristic	Study group, No. (%)	
	Control (n = 121)	Intervention (n = 180)
No. of patients per physician, median (IQR) [range]	5.0 (3.0-7.8) [1-12]	6.0 (3.3-7.0) [1-16]
Age, median (IQR), y	63.1 (51.30-77.51)	64.6 (51.10-75.03)
Sex		
Female	56 (46.3)	74 (41.1)
Male	65 (53.7)	106 (58.9)
Race		
Asian	16 (13.2)	17 (9.4)
Black or African American	9 (7.4)	17 (9.4)
White	66 (54.5)	75 (41.7)
Multiracial or other^a^	30 (24.8)	71 (39.4)
Ethnicity		
Hispanic, Latina, Latino, or of Spanish origin	39 (32.2)	67 (37.2)
Not Hispanic, Latina, Latino, or of Spanish origin	81 (66.9)	111 (61.7)
Not reported	1 (0.8)	2 (1.1)
Comorbidities		
Chronic heart failure	24 (19.8)	28 (15.6)
Chronic kidney disease	25 (20.7)	27 (15.0)
Site		
Jacobs Medical Center	67 (55.4)	116 (64.4)
Hillcrest Medical Center	54 (44.6)	64 (35.6)

^a^ Includes American Indian or Alaska Native, Native Hawaiian or Other Pacific Islander, and unknown race.

Between December 2024 and July 2025, eligible attending physicians managed 400 ED encounters with a diagnosis of sepsis, severe sepsis, or septic shock, of which 99 were excluded due to not meeting CMS criteria for severe sepsis or to having a time of sepsis onset outside the ED. The median number of patients per physician was 5.0 (IQR, 3.0-7.8) in the control group and 6.0 (IQR, 3.3-7.0) in the intervention group. The number of patients per individual physician is described in eTable 2 in Supplement 2.

Physicians in the intervention group had an SEP-1 compliance rate of 82.9% and those in the control group had a compliance rate of 70.1%. Our primary mixed-effects model found that assignment to the intervention group resulted in a 13.0% absolute improvement in SEP-1 compliance (95% CI, 2.5%-23.4%; odds ratio, 2.10 [95% CI, 1.15-3.81]; *P* = .02) with an intraclass correlation coefficient of 0.018, representing minimal clustering by physician. Our secondary analysis of physician-level comparison of compliance rates confirmed these results (*t* = −2.5447; *P* = .02). The higher compliance rate for the sepsis bundle in the intervention group was primarily achieved through increased compliance with the 30-mL/kg crystalloid fluid bolus component. This component allows administration of less than 30 mL/kg when justified by physician documentation (eg, heart failure, kidney failure, concern for fluid overload). As such, Table 2 also reports fluid volume administration greater than 30 mL/kg, which is higher in the intervention group (noncompletion, 3 of 180 [1.7%] vs 16 of 121 [13.2%])) but does not reach statistical significance. The compliance rate over time is visualized in the eFigure in Supplement 2. Final agreement between the LLM and expert human reviewers was 92%, and 276 patients (91.7%) included in our study eventually received a billing diagnosis code for sepsis, severe sepsis, or septic shock. We did not observe any significant differences in 30-day mortality or intensive care unit admission between the intervention and control groups.

**Table 2.** Severe Sepsis and Septic Shock Management Bundle (SEP-1) Compliance Variables

Variable	Study group, No./total No. (%)^a^		OR (95% CI)	*P* value
	Control (n = 121)	Intervention (n = 180)		
SEP-1 compliance				
Overall	82/117 (70.1)	145/175 (82.9)	2.10 (1.15-3.81)	.02
Jacobs Medical Center	44/66 (66.7)	94/114 (82.5)	NA	NA
Hillcrest Medical Center	38/51 (74.5)	51/61 (83.6)	NA	NA
Process step missed				
Blood culture collection	1 (0.8)	4 (2.2)	2.44 (0.17-35.4)	.52
Broad spectrum or other antibiotic administration	6 (5.0)	8 (4.4)	0.89 (0.30-2.64)	.84
Initial lactate level collection	0	0	NA	NA
Crystalloid fluids administration	16 (13.2)	3 (1.7)	0.11 (0.03-0.39)	<.001
Persistent hypotension	5 (4.1)	10 (5.6)	1.36 (0.43-4.30)	.60
Repeat lactate level collection	3 (2.5)	3 (1.7)	0.66 (0.13-3.36)	.62
Repeat volume status	4 (3.3)	2 (1.1)	0.37 (0.01-13.20)	.59
Interventions				
Fluid volume ≥30 mL/kg^b^	38/57 (66.7)	54/73 (74.0)	1.42 (0.67-3.04)	.36
Outcomes				
ICU admission	25 (20.7)	36 (20.0)	0.96 (0.54-1.70)	.89
30-d Mortality	13 (10.7)	25 (13.9)	1.34 (0.65-2.77)	.43
Septic shock	28 (23.1)	34 (18.9)	0.70 (0.40-1.22)	.21

Abbreviations: ICU, intensive care unit; NA, not applicable; OR, odds ratio.

^a^ Per the Centers for Medicare & Medicaid Services, patients can be in-numerator, in-denominator, or out of measure.

^b^ Fluid volume between 6 hours prior to and 3 hours after hypotension or septic shock as defined by SEP-1.

## Discussion

In this study, the use of generative AI to assess quality of sepsis care coupled with active feedback in 2 EDs significantly improved SEP-1 performance, representing the first instance, to our knowledge, of a generative AI tool improving physician adherence to a quality metric in the hospital setting. Increased performance in a publicly reportable quality metric has major implications for hospital reimbursement from CMS. Improvement was associated with enhancing feedback loops through timely targeted education, engaged leadership, and better documentation. In contrast to the status quo, AI-enabled real-time abstraction offers a solution for timely feedback to clinicians to improve quality metric performance. More broadly, this application of generative AI might address the critical limitations of existing quality measurement.

Although SEP-1 compliance increased and a greater percentage of patients with severe sepsis or septic shock received a 30-mL/kg fluid bolus, our intervention did not improve mortality. Several factors may explain this finding. First, as the study was designed for other purposes, our investigation was not powered to detect a mortality difference. Second, while data suggest that sepsis bundles improve patient-centered outcomes, a recent systematic review highlighted that no moderate- or high-level evidence associates SEP-1 compliance with improved survival.^22^ Therefore, the impact of AI-generated near-real-time SEP-1 compliance feedback on patient-centered outcomes similarly remains uncertain. Third, although we observed an increasing trend in administration of the guideline-recommended 30-mL/kg fluid bolus, our study may not have been powered to detect a statistically significant difference in this measure. In addition, higher SEP-1 compliance may reflect improved documentation of appropriate fluid-bolus exemptions rather than changes in actual clinical management. While improved documentation may not directly impact patient care, SEP-1 adherence remains of great interest to health systems, as it is a publicly reportable quality metric with direct financial implications via the CMS Value-Based Purchasing Program, which rewards acute care hospitals with incentive payments for quality metric performance.

More broadly, our investigation demonstrates a technological advancement in the audit and feedback strategy to improve professional performance.^23^ In this, the audit refers to a systematic evaluation of a clinician’s performance based on explicit standards. The results of the audit (feedback) are then given to the clinician in a standardized manner. A recent meta-analysis^24^ found modest improvements using an audit and feedback strategy, with a mean absolute increase in desired practice by 6.2% across a variety of practice locations and quality initiatives. Importantly, the authors found that this strategy was most effective in particular scenarios relevant to our investigation.^24^ For example, greater benefit was found when performance metrics have substantial room for improvement, where feedback is targeted to the individual (not the organization), and where an actionable plan within specific recommendations for improvement are provided. For sepsis care, where many health systems in the US provide feedback only on cases specifically reviewed for SEP-1 submission to CMS, our LLM-assisted audit and feedback strategy may afford physicians with a broader and more timely understanding of their individual performance and targeted areas for improvement.

While our work focused on improving a single process measure, this same conceptual approach could be applied to other quality and safety measures at scale. A shift toward AI-driven quality clinical integration might result in major cost savings and reduced person-hours in reporting such metrics. Moreover, the ability of AI to accurately and contemporaneously extract clinical context from the medical record may facilitate seamless embedding of best practices within the delivery process. Future work may enable the integration of such clinical insight into real-time clinical care, converting the often cumbersome administrative process of quality reporting into clinically meaningful quality care, thus empowering a true learning health system to cross the quality chasm as the National Academy of Medicine originally envisioned 25 years ago.^1,25^

### Limitations

We acknowledge several limitations of our study. First, there were more patient encounters in the intervention group (n = 180) compared with the control group (n = 121) due to variations in ED shift allotment between physicians and an unexpected exit of certain physicians. Second, we conducted our intervention within an academic health system during a 7-month period. External generalizability and long-term sustainability require confirmation. Finally, at both our sites, resident physicians cared for patients and were not part of the feedback system. However, the attending of record was ultimately responsible for patient care and overseeing our resident physician’s therapeutic plan and documentation.

## Conclusions

In this cluster randomized trial of AI-enabled medical record abstraction, near–real-time personalized feedback to attending physicians in the ED resulted in a significant increase in SEP-1 process compliance compared with those who did not receive this. Whether this approach could improve actual clinical management or patient-centered outcomes remains uncertain and requires further investigation. Regardless, these findings suggest that AI-enabled abstraction may ameliorate existing limitations in quality measurement.

## Notes

Supplement 1.
Trial Protocol and Statistical Analysis Plan

Supplement 2. **eTable 1.** Iterative Improvements to the SEP-1 AI System **eTable 2.** Number of SEP-1 Cases by Emergency Department Provider **eFigure.** Monthly SEP-1 Adherence Rate Over Time by Study Arm

Supplement 3.
Data Sharing Statement
