# Supplementary material for: Medical Record Abstraction for Quality Improvement in Sepsis Care Using Artificial Intelligence: A Cluster Randomized Trial
Source: JAMA Netw Open. Author manuscript; Available in PMC 2026 Jul 21. (PMC13306301; doi:10.1001/jamanetworkopen.2026.11885)
Supplement: sup3 — SUPPLEMENT 3. Data Sharing Statement [file NIHMS2191457-supplement-sup3.pdf]

Data Sharing Statement

Boussina. Medical Record Abstraction for Quality Improvement in Sepsis Care Using Artificial Intelligence. *JAMA Netw Open*. Published June 25, 2026.
doi:10.1001/jamanetworkopen.2026.11885

Data
Data available: No
