# Supplementary material for: Medical Record Abstraction for Quality Improvement in Sepsis Care Using Artificial Intelligence: A Cluster Randomized Trial
Source: JAMA Netw Open. Author manuscript; Available in PMC 2026 Jul 21. (PMC13306301; doi:10.1001/jamanetworkopen.2026.11885)
Supplement: sup2 — SUPPLEMENT 2. eTable 1. Iterative Improvements to the SEP-1 AI System eTable 2. Number of SEP-1 Cases by Emergency Department Provider eFigure. Monthly SEP-1 Adherence Rate Over Time by Study Arm [file NIHMS2191457-supplement-sup2.pdf]

Supplementary Online Content

Boussina A, Claire A, Quintero K, et al. Medical record abstraction for quality improvement in sepsis care using artificial intelligence: a cluster randomized trial. *JAMA Netw Open.* 2026;9(5):e2611885. doi:10.1001/jamanetworkopen.2026.11885

eTable 1. Iterative Improvements to the SEP-1 AI System
eTable 2. Number of SEP-1 Cases by Emergency Department Provider
eFigure. Monthly SEP-1 Adherence Rate Over Time by Study Arm

This supplementary material has been provided by the authors to give readers additional information about their work.

© 2026 Boussina A et al. *JAMA Network Open*.

eTable 1. Iterative Improvements to the SEP-1 AI System

Date	Issue	Description of Change
December 13th, 2024	Improper tagging of organ dysfunction for patient with ESRD[1] on dialysis.	Suppression of elevated creatinine for patients with physician/APN/PA documentation before or within 24 hours following presentation of severe sepsis that states that the patient has ESRD[1] and is on hemodialysis or peritoneal dialysis.
February 18, 2025	Improper determination of time of sepsis for patient where sepsis time is specified within note text.	Addition of logic to process free text for explicit mention of the time of sepsis and compare to “time zero” from the clinical criteria.
March 31st, 2025	Improper tagging of mechanical ventilation for a patient not on respiratory support.	Fix of software bug resulting in PEEP/FiO2 sometimes being incorrectly interpreted as mechanical ventilation.

[1]End Stage Renal Disease

© 2026 Boussina A et al. *JAMA Network Open*.

eTable 2. Number of SEP-1 Cases by Emergency Department Provider

**Provider UUID**	**N**
**Intervention**	
8ff4216a-4ead-4f13-a3da-9827210ab47b	16
85ca1e96-bec4-4ff7-8c52-29a07fecd9b0	13
84390d6c-b7f0-4dc7-ad6f-15d5e261adb9	12
0a8abb5a-eb32-4233-95ae-bc5f195a12f1	10
0cea1e62-e1db-4d97-8945-19c4abdf4fda	10
34b5dc5b-47b8-4648-aa22-334d44c3f89d	10
2fd34f10-6d80-4a90-86be-772efe60aef3	9
7ad732ad-205d-41bd-98b4-ca8bc4cf77e5	7
8882850d-2583-43b6-bbbc-1b53791e505d	7
90691b61-75c7-4c93-90de-3b922ac9eca8	7
dea67059-e3d3-43bd-a78a-b73b9c659c1d	7
4821d8d7-813a-4c5a-955a-81cd71553792	6
85ec69bc-6617-436f-af37-c4359fa38609	6
8a5ab13d-f5f2-4097-860c-9f24a07807f5	6
d34b1f79-2da4-4c68-b4eb-b1093376c71b	6
eb100f89-1527-49de-90fe-15cfb0047ee6	6
80f4a001-75cb-4112-8b66-2f6ea4186b66	5
168e54e4-5ccc-4b95-829c-3607b45a5a87	4
410d2483-a101-430d-8610-b8acd057687b	4
5dca1b71-5b2c-41d3-b85a-0e39cc8f8578	4
6ce935df-cd26-4427-bc28-8ed4facf81e5	4
9b3faa75-137f-4c05-a702-aee7cd76f506	4
5a85edd4-8b38-4156-8523-2d6230ad1843	3
78d3c4d6-5588-47b3-853f-8ef80edd72c2	3
957f33b9-adeb-4e0c-b170-2a44e7a03c01	3
164a32b9-9861-4211-8ecb-2918e94970d9	2
a0ce7ea6-92b5-4bbf-b063-218fab43cd9b	2
a41a22f8-752f-4650-b2d8-82b2dc7f8c37	2
e9e0ea2c-9ac8-4478-be11-d6b9236ee70a	1
f2694825-dbf2-45f3-b539-1523e8a76c53	1

© 2026 Boussina A et al. *JAMA Network Open*.

Control	
b3a6bdcd-f58c-4696-ad63-a5192cd10782	12
b6f546d1-2ab2-4ac6-94b9-b9cc30819a85	11
0f35faa9-f64a-43cf-a66a-f06ffd50b8f0	10
87ae64b1-cb2c-4c09-8b56-8797a211bdb5	9
f19e9f2b-3b3a-45e4-a3b8-fd776addc1ba	9
19205b85-195e-4297-9b74-e3855e8cd01a	8
830266a4-4c05-4fba-8c3f-b4497c326e58	8
1ea91d9f-d344-438b-a15c-83f42f6860b0	7
1fc4c03d-3f56-4e6e-8882-ddd423c82ecf	6
9107a805-db04-4a2b-b718-57e006fab24b	5
96e18491-58fd-42c0-9948-68f79bae60e5	5
bca036f8-6574-4741-826e-f87fb73bbedd	5
4d123fe6-f66b-4f9d-8f4a-9da82f299716	4
0b0be561-662e-433b-94e4-1fd18ef8d195	3
bb9a090a-bdad-433d-94da-4c8f3288f337	3
d652b146-9b7f-4b9f-8a84-2241699bc721	3
f055d1b3-bcc5-49ca-92ce-66753965868d	3
fa475330-d548-46f5-aee9-63ecc2b8b504	3
faa706db-999a-421f-98da-1cdb10e30a34	3
32426784-5ca6-491b-9c7a-6cbb4013735c	1
4b0d8cc3-70aa-4a37-9234-d5bc59323093	1
a2ffe833-7ef9-40b5-a76b-fc70e03e505c	1
af1bc933-d1d2-44e0-89e5-e0e22aec21e0	1

© 2026 Boussina A et al. *JAMA Network Open*.

eFigure. Monthly SEP-1 Adherence Rate Over Time by Study Arm

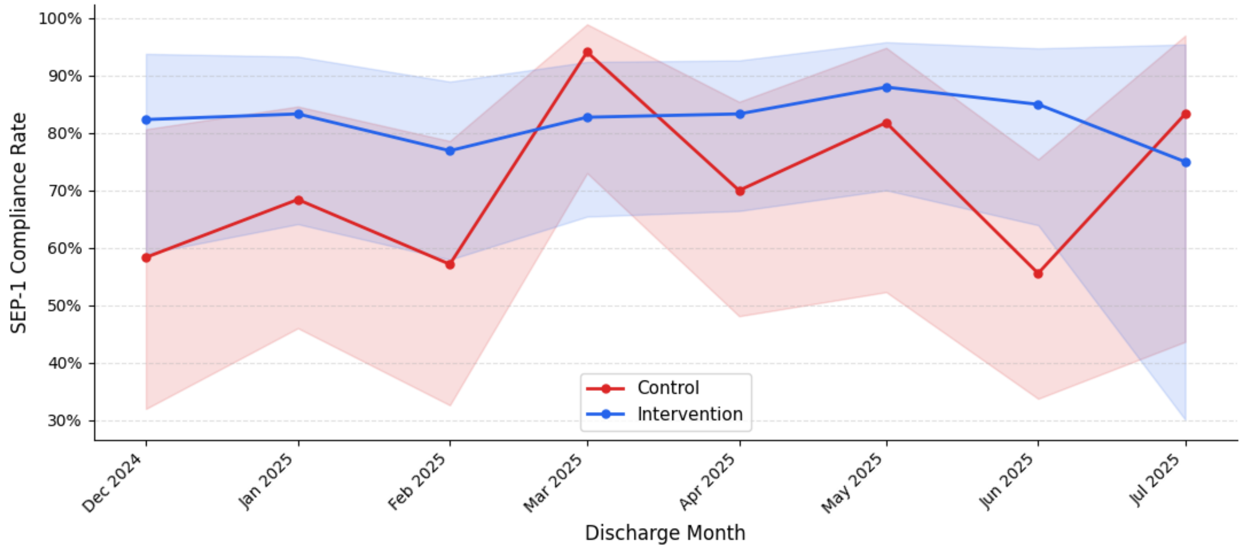

Plot of compliance by study arm for each month of the study. Wilson 95% confidence intervals are displayed as shaded bands.

© 2026 Boussina A et al. *JAMA Network Open*.
