# Supplementary material for: Medical Record Abstraction for Quality Improvement in Sepsis Care Using Artificial Intelligence: A Cluster Randomized Trial
Source: JAMA Netw Open. Author manuscript; Available in PMC 2026 Jul 21. (PMC13306301; doi:10.1001/jamanetworkopen.2026.11885)
Supplement: sup1 — SUPPLEMENT 1. Trial Protocol and Statistical Analysis Plan [file NIHMS2191457-supplement-sup1.pdf]

Statistical Analysis Plan

Title: Impact of Automated Sepsis Metric Evaluation on Provider Performance: A Cluster Randomized Quality Improvement Study

Protocol Version: 1.0
SAP Version: 1.0
Institution: UC San Diego Health, Department of Emergency Medicine
ACQUIRE Project #: 1403 (Kuali #178)
Principal Investigators: Aaron Boussina, PhD; Gabriel Wardi, MD
Statistician: Sonia Jain, PhD

Table of Contents

1. Abbreviations
2. Study Overview
3. Objectives and Hypotheses
4. Study Population
5. Randomization and Blinding
6. Intervention and Comparator
7. Outcome Definitions
8. Analysis Populations
9. Sample Size and Power
10. Statistical Methods — Primary Outcome
11. Statistical Methods — Secondary Outcomes
12. Sensitivity Analyses
13. Software and Significance Threshold

1. Abbreviations

Abbreviation	Definition
A&F	Audit and Feedback
APP	Advanced Practice Provider
CI	Confidence Interval
CMS	Centers for Medicare & Medicaid Services
ED	Emergency Department
HVBP	Hospital Value-Based Purchasing Program
ICC	Intraclass Correlation Coefficient
ICD	International Classification of Diseases
ICU	Intensive Care Unit
IQR	Interquartile Range
ITT	Intent-to-Treat
LLM	Large Language Model
OR	Odds Ratio
QI	Quality Improvement
SAP	Statistical Analysis Plan
SEP-1	Severe Sepsis and Septic Shock Management Bundle (CMS measure)
SS/SS	Severe Sepsis / Septic Shock
UCSD	University of California, San Diego

2. Study Overview

2.1 Design

This is a prospective, single-blind, unstratified cluster randomized quality improvement (QI) study. Randomization is at the level of the attending physician (the cluster unit); outcomes are measured at the level of the individual patient encounter.

2.2 Setting

Two academic emergency departments (EDs) within the UC San Diego Health system:

- **La Jolla Campus ED** (UCSD Jacobs Medical Center)
- **Hillcrest Campus ED** (UCSD Medical Center - Hillcrest)

Combined annual ED census: approximately 90,000 visits. The study was conducted under the auspices of the UCSD ACQUIRE Committee (Project #1403), which determined that the project does not constitute human subjects research under 45 CFR 46 or 21 CFR 56. The study is reported in accordance with the SQUIRE 2.0 guidelines for quality improvement reporting.

2.3 Study Period

Enrollment: December 2024 through July 2025 (approximately 7 months).

2.4 Background and Rationale

SEP-1 is a publicly reportable, 63-step CMS quality measure for severe sepsis and septic shock (SS/SS) that was incorporated into the Hospital Value-Based Purchasing Program in fiscal year 2024. Standard CMS reporting requires manual abstraction of a maximum of 20 cases per month, delivered to the health system approximately 3-4 months after discharge. This cadence precludes timely individual provider feedback. An LLM system previously validated to achieve >90% agreement with expert human abstractors for SEP-1 abstraction (Boussina et al., NEJM AI, 2024) enables automated, near-real-time evaluation of every eligible ED encounter. The pre-intervention SEP-1 compliance rate for the UCSD ED was approximately 55% in FY2023-24. Providing individualized, timely feedback informed by this system constitutes the experimental intervention.

---

3. Objectives and Hypotheses

3.1 Primary Objective

To determine whether near-real-time, individualized feedback on SEP-1 compliance, delivered by automated LLM abstraction at the time of patient discharge, improves overall SEP-1 bundle compliance compared with standard practice over the study period.

Primary Hypothesis:
H_0: The proportion of patient encounters with full SEP-1 compliance does not differ between providers randomized to automated feedback and providers receiving standard care.
H_1: The proportion of patient encounters with full SEP-1 compliance is higher among providers randomized to automated feedback.

3.2 Secondary Objectives

1. To assess agreement between the LLM's SEP-1 determination and that of expert human reviewers on in-measure cases.
2. To compare 30-day all-cause mortality of patients with SS/SS between the intervention and control provider groups.
3. To compare ICU admission rates of ED patients with SS/SS between the intervention and control provider groups.
4. To compare compliance rates for each individual component of the SEP-1 bundle between the intervention and control groups.

---

4. Study Population

4.1 Cluster-Level (Provider) Inclusion Criteria

- Attending physician (MD) with active clinical privileges in the UCSD ED (La Jolla or Hillcrest)
- Working ≥3 ED shifts per month at the time of study initiation
- Primary attending of record for at least one eligible patient encounter during the study period

4.2 Cluster-Level (Provider) Exclusion Criteria

- Providers who cease all ED clinical activities at both study sites prior to any patient encounter during the study period

Note: Providers who leave or reduce shifts after randomization remain in the analysis per the intent-to-treat (ITT) principle. Providers who joined the clinical staff after randomization are not eligible for inclusion.

4.3 Encounter-Level (Patient) Inclusion Criteria

- Patient encounter with a clinical diagnosis of severe sepsis or septic shock
- Time zero of SS/SS onset, as defined by CMS SEP-1 criteria, occurring under the care of a study-enrolled attending physician
- Encounter occurs during the pre-specified study period

Note: Clinical encounter diagnosis is used to identify eligible encounters at the time of discharge, prior to availability of billing codes.

4.4 Encounter-Level (Patient) Exclusion Criteria

- No time zero or a time zero occurring outside of the ED setting
- Attending of record is not a study-enrolled physician

---

5. Randomization and Blinding

5.1 Unit of Randomization

Individual attending physicians constitute the cluster unit of randomization.

5.2 Randomization Procedure

All eligible attending physicians are randomized in a 1:1 ratio to intervention or control using simple (unstratified) randomization. No stratification by site, years of experience, baseline SEP-1 compliance, or other covariates is performed.

5.3 Blinding

The study is single-blind. Physicians in the control arm are not informed of the study allocation of other providers or of the fact that all eligible encounters are being evaluated by the LLM. Physicians in the intervention arm are aware of their assignment. Members of the analysis team are not blinded to allocation during outcome adjudication but statistical analyses are pre-specified prior to outcome analysis in this plan.

---

6. Intervention and Comparator

6.1 Intervention (Experimental Arm)

Attending physicians randomized to the intervention receive the following:

Per-encounter feedback: Following each eligible patient encounter, the LLM system evaluates the entire medical record for SEP-1 compliance at the time of discharge. A standardized email is generated within 24 hours of discharge containing:

1. For compliant encounters: a congratulatory notification and a brief reminder of UCSD sepsis policy.
2. For non-compliant encounters: a case summary identifying the specific SEP-1 component(s) not met, with targeted guidance on corrective care or documentation. For select cases, supplemental verbal feedback is provided by the Medical Director of the UCSD Sepsis Program.

6.2 Control (Standard Practice Arm)

Attending physicians randomized to control receive standard institutional feedback, consisting of:

- Case-level feedback only when their patient is selected for formal CMS reporting (up to 20 cases per month across all ED and inpatient settings, sampled randomly, typically delivered 3-4 months after discharge).
- On average, most providers in this arm receive feedback on 1-2 cases annually.

The LLM system evaluates all eligible encounters regardless of arm assignment but feedback is withheld from control-arm providers.

---

7. Outcome Definitions

7.1 Primary Outcome

SEP-1 compliance (encounter level): Binary indicator (1 = in-numerator; 0 = not in-numerator) per CMS SEP-1 criteria, as adjudicated by three experienced study team reviewers with independent LLM evaluation. Where LLM and expert reviewer disagree, a physician expert with SEP-1 abstraction expertise adjudicates the case. The final determination serves as the outcome variable.

7.2 Secondary Outcomes

1. **LLM-expert agreement (encounter level):** Binary indicator of whether the LLM's initial SEP-1 determination (pass/fail) matches the final expert human reviewer determination, expressed as a percentage. Computed for all in-measure encounters.

2. **30-day all-cause mortality (encounter level):** Binary indicator (1 = death within 30 days of ED encounter; 0 = alive at 30 days), ascertained from the electronic health record (Epic Clarity).

3. **ICU admission (encounter level):** Binary indicator (1 = ICU admission during the index hospitalization; 0 = no ICU admission).

4. **Individual SEP-1 component non-compliance (encounter level):** Separate secondary outcomes for (1) Blood Culture Collection, (2) Broad Spectrum or Other Antibiotic Administration, (3) Initial Lactate Level Collection, (4) Crystalloid Fluids Administration, (5) Persistent Hypotension, (6) Repeat Lactate Level Collection, (7) Repeat Volume Status.

---

8. Analysis Populations

8.1 Intent-to-Treat (ITT) Population — Primary

The ITT population includes all patient encounters meeting eligibility criteria (Sections 4.3 and 4.4) attended by randomized physicians who managed at least one eligible patient encounter during the study period. This is the primary analysis population.

---

9. Sample Size and Power

9.1 Primary Calculation

Sample size was estimated for the encounter-level primary outcome under the following assumptions:

- **Baseline compliance rate (control arm):** 65% (derived from FY2022-23 institutional SEP-1 performance)
- **Minimum detectable difference:** 15 percentage points absolute increase in compliance in the intervention arm (i.e., 80% vs. 65%)

- **Power:** 80% (two-sided)
- **Type I error:** $\alpha = 0.05$
- **Clustering adjustment:** Given the cluster-randomized design with outcomes nested within physicians, the effective sample size is reduced by the design effect (DEFF = 1 + (m − 1) × ICC, where m is the mean number of encounters per physician). At the time of sample size calculation, ICC was assumed to be low, yielding a DEFF close to 1.0 for the expected encounter counts. Under these parameters, a minimum of 300 total in-measure patient encounters was specified.

---

10. Statistical Methods — Primary Outcome

10.1 Model Specification

Because randomization occurred at the physician level and the primary outcome is measured at the encounter level, the primary analysis accounts for clustering of encounters within physicians. The primary analysis uses a **mixed-effects logistic regression** model with the following specification:

$$\text{logit}(P(\text{SEP-1 compliance} = 1)_{ij}) = \beta_0 + \beta_1(\text{Intervention})_j + u_j$$

Where:

- i indexes patient encounter within physician j
- β_1 is the fixed effect for intervention assignment (coded 1 = intervention, 0 = control)
- $u_j \sim N(0, \sigma^2_u)$ is a physician-specific random intercept capturing within-physician correlation of outcomes across encounters

10.2 Reported Quantities

From the primary model:

- **Odds Ratio (OR)** for intervention vs. control with 95% CI and two-sided p-value
- **Intraclass Correlation Coefficient (ICC):** representing the proportion of total encounter-level variance attributable to between-physician clustering
- **Average Marginal Effect (AME):** the model-based estimate of the absolute difference in the probability of SEP-1 compliance (intervention minus control), averaged over the observed distribution of random effects. The AME and its 95% CI constitute the primary effect size estimate reported alongside the OR.

10.3 Covariates

The primary model includes only the intervention indicator and physician random intercept. No baseline covariates are included in the primary model, consistent with the unstratified randomization design.

---

11. Statistical Methods — Secondary Outcomes

All secondary outcomes are analyzed using the same mixed-effects model framework as the primary outcome, with the respective secondary outcome as the dependent variable, intervention group as fixed effect, and physician random intercept. Each secondary outcome is analyzed in a separate model.

11.1 LLM-Expert Agreement

LLM-expert agreement is a simple percentage computed over all in-measure encounters and reported as a point estimate with exact binomial 95% CI. No regression model is used for this outcome because it is not a function of provider allocation.

11.2 30-Day Mortality

Mixed-effects logistic regression with 30-day mortality (binary) as dependent variable, intervention arm as fixed effect, physician random intercept. OR, 95% CI, and p-value reported.

11.3 ICU Admission

Mixed-effects logistic regression with ICU admission (binary) as dependent variable, intervention arm as fixed effect, physician random intercept. OR, 95% CI, and p-value reported.

11.4 Individual SEP-1 Component Non-Compliance

For each of the six SEP-1 components, a mixed-effects logistic regression is fit with the binary non-compliance indicator for that component as the dependent variable, intervention arm as fixed effect, physician random intercept. ORs, 95% CIs, and p-values reported for each component.

---

12. Sensitivity Analyses

As a secondary analysis, SEP-1 compliance is summarized at the physician level as the proportion of eligible encounters for each provider that were SEP-1-compliant. A **two-sample Student's t-test** is used to compare mean physician-level compliance rates between the intervention and control groups. This analysis treats each physician as a single observation, thereby eliminating within-physician correlation and providing a corroborative estimate of the intervention effect.

---

13. Descriptive Statistics

All analyses are preceded by a descriptive summary of the study population. Variables are summarized as follows:

- Continuous variables: median and interquartile range (IQR) [minimum, maximum] or mean ± standard deviation, as appropriate for the distribution
- Categorical variables: count (N) and percentage
- Group comparisons in the descriptive table are for characterization purposes only and do not constitute inferential tests

Variables to be described, stratified by intervention and control arm:

- Median (IQR) cases per physician
- Age (median, IQR)
- Sex
- Race
- Ethnicity
- Presence of chronic heart failure
- Presence of chronic kidney disease
- Site of care (La Jolla; Hillcrest)
- Septic shock vs. severe sepsis without shock

---

14. Software and Significance Threshold

All analyses are conducted using **R version 4.5.2** or later.

Key packages:

- `lme4` (version 1.1.38 or later): mixed-effects logistic regression
- `performance` (version 0.16.0 or later): ICC estimation
- `marginaleffects` or `lme4`-based methods: average marginal effect estimation

A two-sided α = 0.05 is considered statistically significant for the primary outcome. All confidence intervals are 95% unless otherwise noted.

Appendix A: Intraclass Correlation Coefficient Interpretation

The ICC for the primary mixed-effects model represents the proportion of total unexplained variance in SEP-1 compliance attributable to between-physician differences. An ICC near zero indicates that encounters within a physician are not substantially more similar to each other than to encounters from other physicians, and that the clustering adjustment has minimal impact on estimates compared to a standard logistic regression. An ICC > 0.10 indicates substantial physician-level clustering that meaningfully affects inference.
